# Low-dose IL-2 shapes a tolerogenic gut microbiota that improves autoimmunity and gut inflammation

**DOI:** 10.1172/jci.insight.159406

**Published:** 2022-09-08

**Authors:** Nicolas Tchitchek, Otriv Nguekap Tchoumba, Gabriel Pires, Sarah Dandou, Julien Campagne, Guillaume Churlaud, Gwladys Fourcade, Thomas W. Hoffmann, Francesco Strozzi, Camille Gaal, Christophe Bonny, Emmanuelle Le Chatelier, Stanislav Dusko Erlich, Harry Sokol, David Klatzmann

**Affiliations:** 1 Sorbonne Université, INSERM, Immunology-Immunopathology-Immunotherapy (i3), Paris, France.; 2 AP-HP, Hôpital Pitié-Salpêtrière, Biotherapy (CIC-BTi) and Inflammation-Immunopathology-Biotherapy Department (i2B), Paris, France.; 3 Micalis Institute, Institut National de la Recherche Agronomique (INRA), AgroParisTech, Univ Paris-Saclay, Jouy-en-Josas, France.; 4 Sorbonne University-UPMC Univ Paris 06, INSERM ERL 1157, Avenir Team Gut Microbiota and Immunity, UMR 7203, Paris, France.; 5 Enterome, Paris, France.; 6 MetaGenoPolis, INRA, Université Paris-Saclay, Jouy-en-Josas, France.; 7 Sorbonne Université, Ecole Normale Supérieure, CNRS, INSERM, AP-HP, Laboratoires des Biomolécules (LBM), Paris, France.; 8 AP-HP, Hôpital Saint Antoine, Department of Gastroenterology and Inflammation-Immunopathology-Biotherapy Department (i2B), Paris, France.

**Keywords:** Autoimmunity, Therapeutics, Autoimmune diseases, Bioinformatics, Immunotherapy

## Abstract

Gut microbiota dysbiosis is associated with inflammatory bowel diseases and with cardiometabolic, neurological, and autoimmune diseases. Gut microbiota composition has a direct effect on the immune system, and vice versa, and it has a particular effect on Treg homeostasis. Low-dose IL-2 (IL-2_LD_) stimulates Tregs and is a promising treatment for autoimmune and inflammatory diseases. We aimed to evaluate the impact of IL-2_LD_ on gut microbiota and correlatively on the immune system. We used 16S ribosomal RNA profiling and metagenomics to characterize gut microbiota of mice and humans treated or not with IL-2_LD_. We performed fecal microbiota transplantation (FMT) from IL-2_LD_–treated to naive recipient mice and evaluated its effects in models of gut inflammation and diabetes. IL-2_LD_ markedly affected gut microbiota composition in mice and humans. Transfer of an IL-2–tuned microbiota by FMT protected C57BL/6J mice from dextran sulfate sodium–induced colitis and prevented diabetes in NOD mice. Metagenomic analyses highlighted a role for several species affected by IL-2_LD_ and for microbial pathways involved in the biosynthesis of amino acids, short-chain fatty acids, and L-arginine. Our results demonstrate that IL-2_LD_ induced changes in gut microbiota that are involved in the immunoregulatory effects of IL-2_LD_ and suggest a crosstalk between Tregs and gut microbiota. These results provide potentially novel insight for understanding the mode of action of Treg-directed therapies.

## Introduction

Autoimmune diseases intrinsically reveal dysregulation of the balance between regulatory and effector immune responses, hence Treg insufficiency (1, 2). Low-dose IL-2 (IL-2_LD_) expands and activates Tregs, and thus has a very broad potential for the treatment of numerous autoimmune, inflammatory, or allergic diseases (2). Furthermore, IL-2_LD_ has pleiotropic effects that can be beneficial in autoimmune diseases, notably the inhibition of the differentiation of naive CD4 cells into proinflammatory Th17 and T follicular helper (Tfh) cells (3, 4). Given that the therapeutic potential of IL-2 is currently being investigated in many autoimmune diseases, understanding its mode of action is of prime importance for rational drug development.

Treg cells are usually defined in humans as CD4^+^Foxp3^+^CD25^+^CD127^lo^ cells, although some Tregs may express a low level of CD25, and as CD4^+^Foxp3^+^ cells in mice. Their main role is to control self-tolerance and inflammation. Indeed, experimental ablation of Tregs immediately triggers severe inflammation, including of the gut, and the development of multiorgan autoimmune diseases (5). Tregs also have important roles in tissue regeneration (6), including in the intestine. IL-2 is the nonredundant key cytokine for the differentiation, survival, and function of Tregs. Mutations of the IL-2 signaling pathway in humans and mice are associated with systemic inflammation linked to Treg deficiencies (2). Tregs meet their metabolic requirements by utilizing fatty acids and pyruvate oxidation, in contrast to effector T cells, which mainly rely on glycolysis.

The composition of gut microbiota is linked to human health and diseases and is in a dynamic interplay with the immune system (7–10). Gut microbiota composition has a direct effect on the immune environment and vice versa (9, 10). Many factors — including diet (11), environmental exposure (12), and antibiotics (13) — can modify microbiota composition, possibly leading to altered immune homeostasis (14, 15) and secondarily to disease induction. The effects of gut microbiota on immune regulation are in part linked to Treg generation and proliferation in Peyer patches and mesenteric lymph nodes (16, 17). Treg homeostasis in the intestine is mainly controlled by commensal microbial metabolism (18, 19). Tregs from Peyer patches and intestinal mucosa participate in the regulation of intestinal inflammation (20, 21). Thus, as IL-2_LD_ expands and activates Tregs at a systemic but also tissue level, it has the potential to affect gut microbiota composition.

Using 16S ribosomal RNA gene and metagenomic sequencing, we demonstrated that IL-2_LD_ affected gut microbiota composition in mice and humans. Moreover, we showed that gut microbiota from IL-2_LD_–treated animals could be efficiently transplanted to recipient mice that were then protected from gut inflammation and diabetes, demonstrating that the immunoregulatory effects of IL-2_LD_ are in part mediated by gut microbiota modulation.

## Results

### IL-2_LD_ expands Tregs and protects against autoimmunity and gut inflammation.

**Figure 1:**
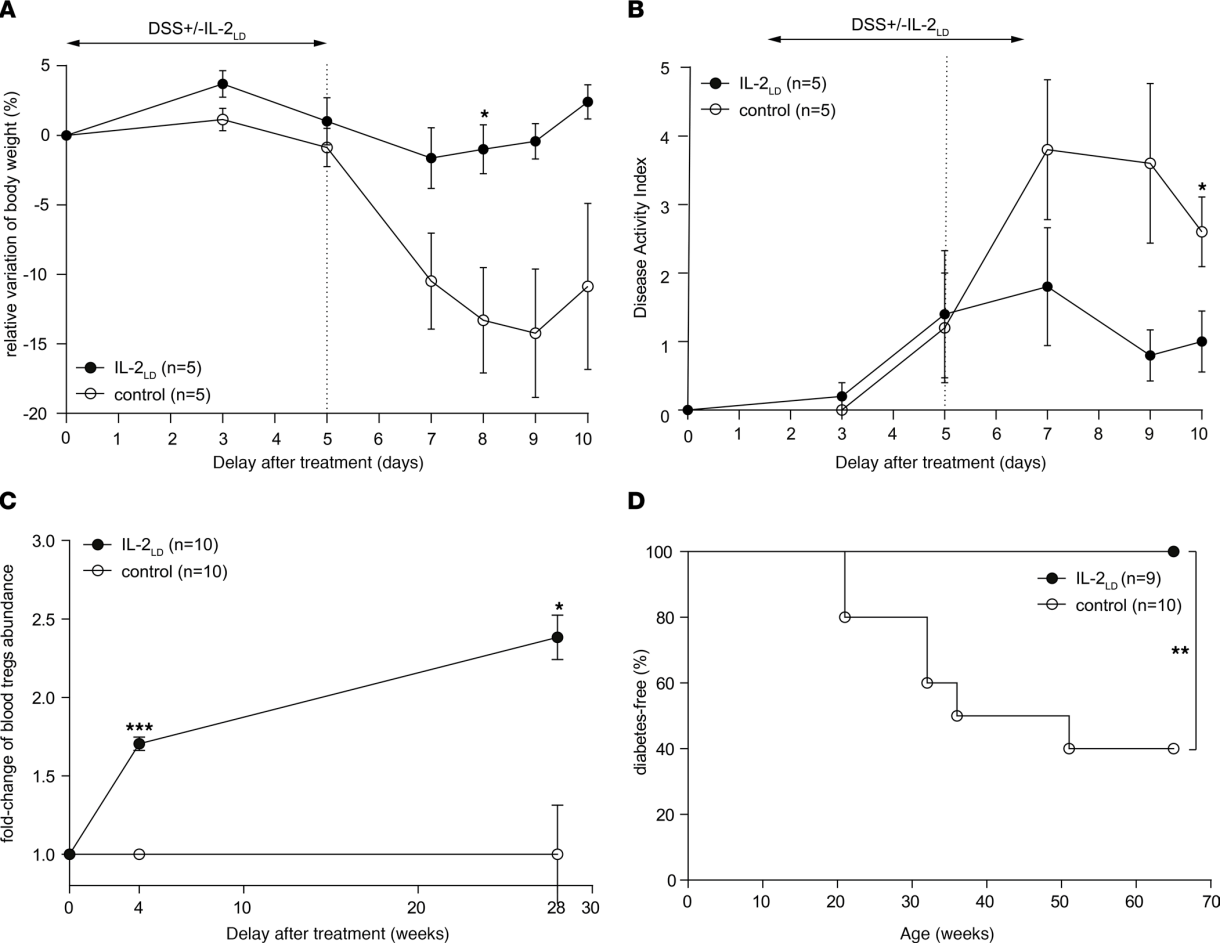
IL-2_LD_ protects from autoimmunity. (**A** and **B**) C57BL/6J female mice were treated or not with IL-2_LD_ by means of intraperitoneal injections of IL-2 during 5 consecutive days. Dextran sulfate sodium (DSS) was administered in drinking water during the same 5 days. The body weight measurements expressed as percentages relative to baseline (**A**) and the colitis disease activity index (**B**) were evaluated at baseline and thereafter. (**C** and **D**) NOD female mice were treated or not with IL-2_LD_ by means of IL-2–producing or luciferase-producing AAV vectors. (**C**) Fold changes of blood Tregs at baseline and after treatment. (**D**) Kaplan-Meier estimators of diabetes onset in NOD mice. Two-tailed Student’s *t* test was used to compare measurements in treated and untreated mice at each time point. The χ² test was used to compare the Kaplan-Meier estimators. The Holm-Bonferroni adjustment was used for multiple-comparison correction. Statistical significances are denoted as follows: *P < 0.05; **P < 0.01; ***P < 0.001. For each biological condition, the total number of mice used is provided in parentheses.

IL-2_LD_ has been shown to prevent and/or treat numerous autoimmune and inflammatory diseases (2). IL-2_LD_ is defined as a dosage that preferentially stimulates Tregs over effector T cells, which corresponds to doses of around 50,000 IU in mice and around 1 to 3 MIU in humans (2). We investigated the immunoregulatory effects of IL-2_LD_ in an inflammatory colitis model induced by the administration of dextran sulfate sodium (DSS) in the drinking water of C57BL/6J mice. At the time of DSS administration, we treated the mice with IL-2_LD_ intraperitoneal injections daily for 5 days. Such treatment expands and activates Tregs systemically (2), including in the colon (22) and lamina propria (23). It led to significantly lower gut inflammation as evidenced by a much-reduced weight loss (Figure 1A) and disease activity index (Figure 1B) compared with controls.

We also investigated the effects of IL-2_LD_ on the prevention of spontaneous diabetes in NOD mice. In this model, diabetes occurs from around 20 to more than 50 weeks of age according to the experimental conditions, including animal housing. Thus, to obtain a long-term stimulation of Tregs without the need for potentially stressful daily injections, we administered IL-2 by means of a single injection of an IL-2–producing adeno-associated virus (AAV) vector (24) that allows long-term production of IL-2. The dose of vector administered was titrated to obtain Treg activation and expansion similar to what is observed with daily intraperitoneal injections of IL-2_LD_ and without stimulation of effector T cells (24). It is noteworthy that, in the pancreas, this treatment expands Tregs without effects on effector T cells, NK cells, and CD8^+^ cells (Supplemental Figure 1; supplemental material available online with this article; https://doi.org/10.1172/jci.insight.159406DS1). Thus, under the conditions used, the AAV–IL-2 treatment can be likened to an IL-2_LD_ treatment by daily injections in terms of Treg effects (Figure 1C). As a control for this procedure, we similarly administered a luciferase-producing AAV vector. Control NOD mice had a 60% rate of diabetes occurrence, while those treated with IL-2 were fully protected from diabetes (Figure 1D).

### IL-2_LD_ affects gut microbiota composition in mice.

To determine whether IL-2_LD_ supplementation induces changes in gut microbiota composition, we first performed 16S rRNA sequencing on feces from 4-week-old NOD and 6-week-old BALB/c mice treated with IL-2 by means of an AAV injection. Because NOD mice have a genetically determined low expression of the *Il-2* gene (25), we hypothesized that this treatment could have a more pronounced effect on their gut microbiota compared with BALB/c mice.

**Figure 2:**
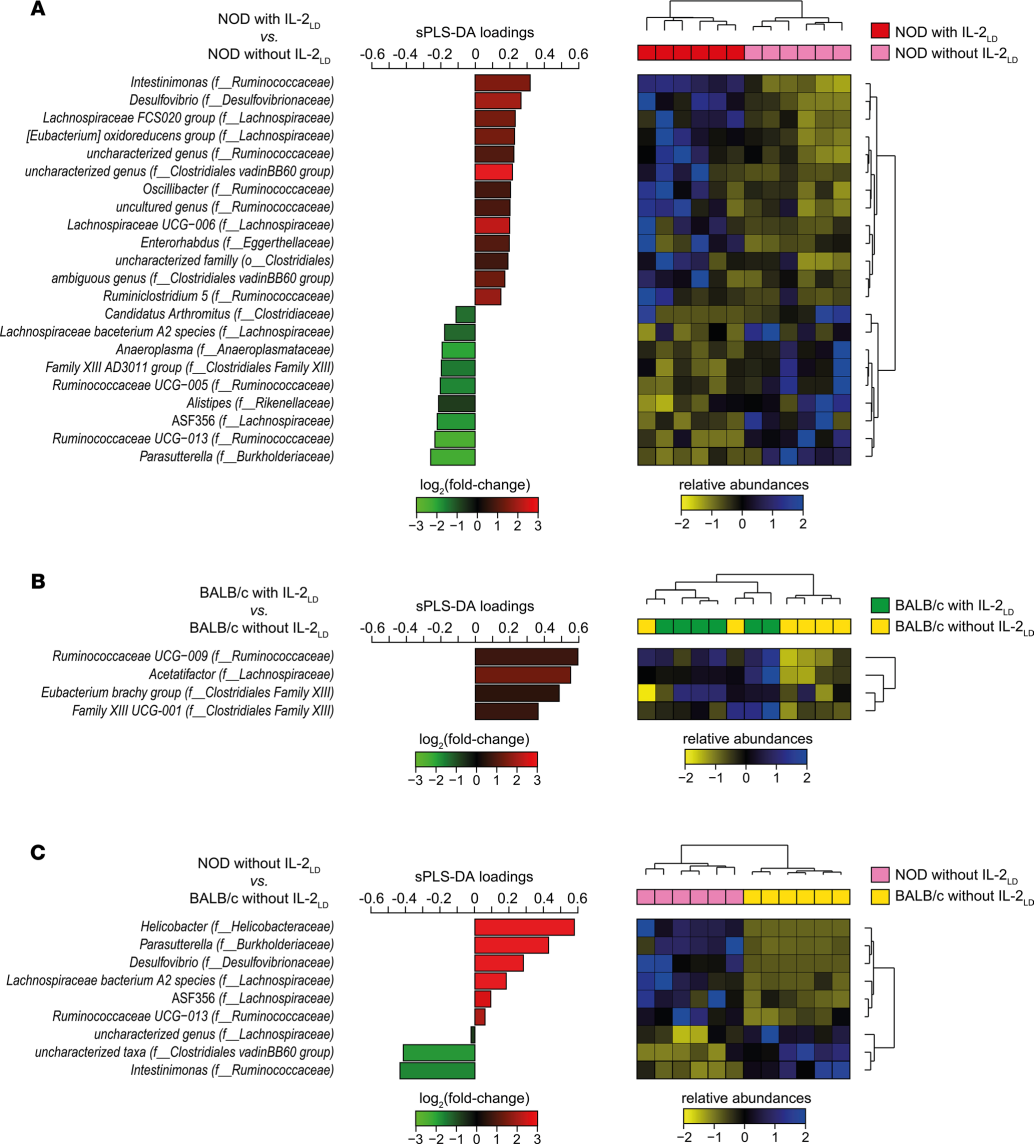
IL-2_LD_ affects gut microbiota composition in mice. NOD and BALB/c mice were treated or not with IL-2_LD_ by means of IL-2–producing or luciferase-producing AAV vectors. Profiling of their gut microbiota, collected 30 days after treatment, was performed using 16S rRNA sequencing. (**A** and **B**) Comparisons were made between NOD mice and BALB/c mice treated or not with IL-2_LD_. (**C**) An additional comparison was made between NOD mice and BALB/c mice without IL-2_LD_ to comprehend the gut microbial specificities associated with these mouse backgrounds. For each comparison, sPLS-DA was conducted to identify the gut microbial taxa best able to discriminate the conditions. The contribution of each taxon identified by sPLS-DA was represented using a horizontal bar of length proportional to its sPLS-DA loading. Horizontal bars were gradient-colored based on the log_2_ of the taxa abundance fold-changes relative to the reference group. For each analysis, a heatmap of relative abundances combined with unsupervised hierarchical clustering was used to evaluate the capacity of the list of taxa to discriminate the conditions. The family (f__) or order (o__) associated with the taxa identified by sPLS-DA is indicated in parentheses.

The IL-2_LD_ treatment markedly modified gut microbiota composition in the feces of NOD mice collected on day 30 after the AAV injection. These NOD mice were 8 weeks old at this time point — long before diabetes onset, which did not occur before 20 weeks in our NOD mouse colony. The abundances of 22 taxa were significantly modified in IL-2_LD_–treated NOD mice compared with controls, 13 taxa being upregulated and 9 taxa being downregulated (Figure 2A). The heatmap of the relative abundance of these 22 taxa, combined with unsupervised hierarchical clustering, highlighted that IL-2_LD_ induced modifications in the gut microbiota composition of NOD mice that allowed a perfect separation of IL-2_LD_–treated mice from untreated mice (Figure 2A).

As hypothesized, the gut microbiota composition was less affected by the IL-2_LD_ treatment in BALB/c mice. Only 4 taxa were significantly modified in IL-2_LD_–treated mice compared with controls (Figure 2B). In line with this observation, the heatmap of relative taxa abundances combined with unsupervised hierarchical clustering outlined that IL-2_LD_ induced gut microbiota modifications that did not allow a perfect separation of IL-2_LD_–treated mice from untreated mice (Figure 2B).

These differences between the changes in microbiota induced by IL-2_LD_ in NOD and BALB/c mice prompted us to compare their gut microbiota composition before treatment. We observed remarkable differences for these mice raised in the same animal facility and fed with the same chow (Figure 2C). A heatmap of relative taxa abundances combined with unsupervised hierarchical clustering allowed a perfect separation of NOD and BALB/c mice (Figure 2C). Among the 22 taxa dysregulated by IL-2_LD_ in NOD mice, *Intestinimonas* was enriched in BALB/c compared with NOD mice and was enriched by IL-2_LD_ in NOD mice. Reciprocally, *Parasutterella*, *Ruminococcaceae UCG−013*, and *ASF356* were less represented in BALB/c compared with NOD mice and were reduced by IL-2_LD_ in NOD mice (Supplemental Figure 2).

Thus, IL-2_LD_ treatment, which confers protection against autoimmunity, was associated with gut microbiota changes in two distinct genetic backgrounds. Moreover, the gut microbiota of NOD mice, which have a genetically determined low IL-2 production (25), was the more affected.

### IL-2_LD_–tuned gut microbiota can be transplanted and confer protection from autoimmunity and gut inflammation.

**Figure 3:**
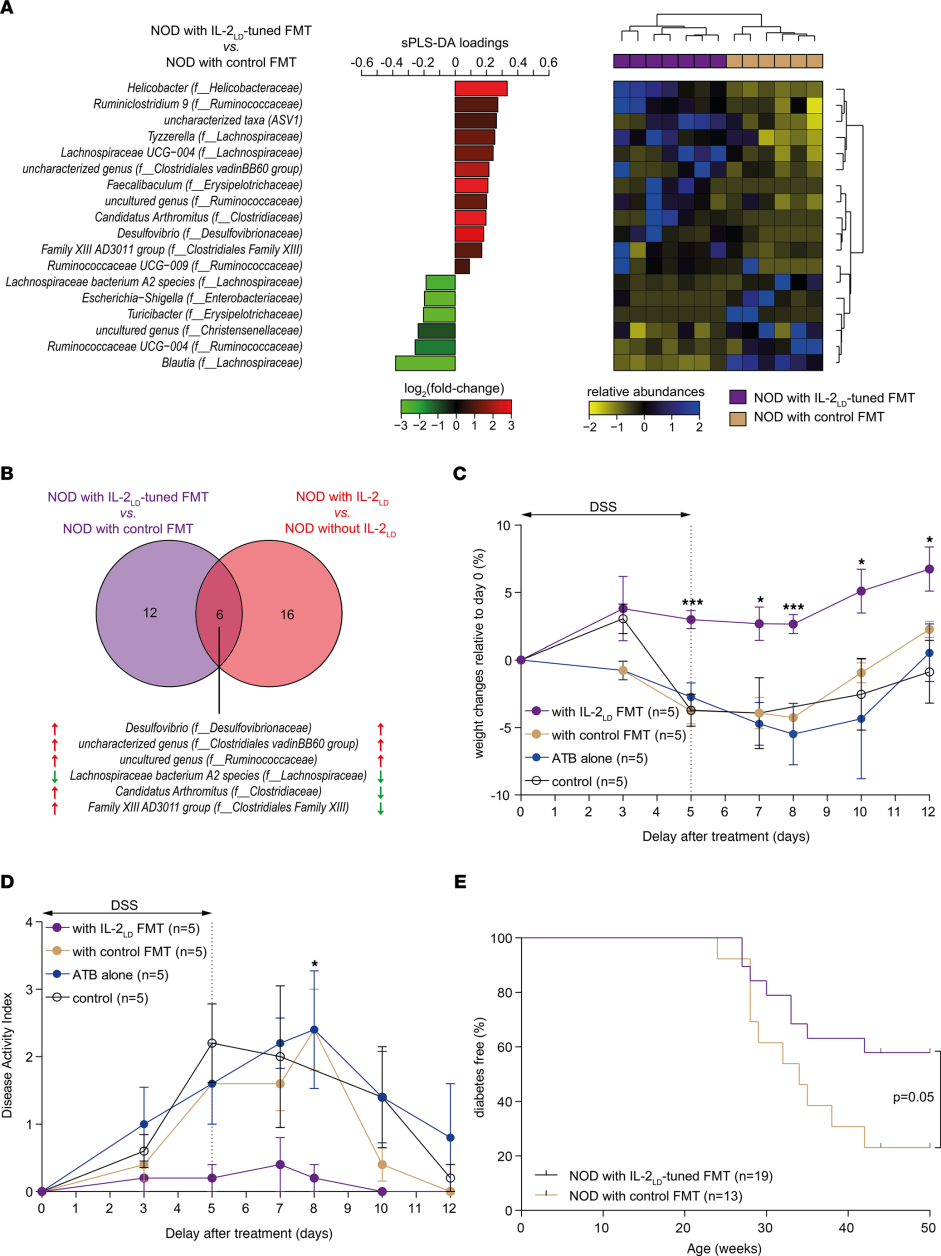
IL-2_LD_–tuned gut microbiota protects from autoimmunity. The microbiota of IL-2_LD_–treated NOD mice was transferred to recipient NOD mice treated by antibiotics (ATB) for 14 days before fecal microbiota transplantation (FMT). NOD mice receiving FMT from untreated NOD mice were used as controls. 16S rRNA sequencing was used to profile gut microbiota at day 30 after treatment. (**A**) sPLS-DA was conducted to identify taxa separating NOD mice with FMT IL-2_LD_ from NOD mice with control FMT. The contribution of each taxon identified by sPLS-DA was represented using a horizontal bar of length proportional to its sPLS-DA loading. A heatmap of relative abundances combined with unsupervised hierarchical clustering was used to evaluate the capacity of the list of taxa to discriminate the conditions. (**B**) Venn diagram showing the overlap between the lists of taxa affected by IL-2_LD_ and IL-2_LD_ FMT treatments relative to their control groups. (**C** and **D**) In an independent experiment, C57BL/6J mice were treated with IL-2_LD_. Five weeks later, fresh stools from these mice were collected and orally administered to 7-week-old female C57BL/6 mice previously treated daily for 14 days with ATB. DSS was administered in drinking water for 5 days after transplantation to trigger colitis. Mouse body weight change (**C**) and disease activity index (**D**) were evaluated until day 12. (**E**) In an independent experiment, 4-week-old female NOD mice were treated with IL-2_LD_ or without IL-2_LD_. Five weeks later, fresh stools from these mice were collected and orally administered to 6-week-old female NOD mice previously treated daily for 14 days with ATB. Diabetes onset was screened for during the experiment. Student’s *t* test was used to compare measurements in treated and untreated mice at each time point. The Holm-Bonferroni adjustment was used for multiple-comparison correction. Statistical significances were reported as follows: *P < 0.05; ***P < 0.001. IL-2_LD_ was administered or not to mice by means of IL-2–producing AAV. For each biological condition, the total number of mice used is provided in parentheses.

Given that IL-2_LD_ modifies gut microbiota composition, which is in interplay with the immune system, we hypothesized that an IL-2_LD_–tuned microbiota could participate in the immunoregulatory effects of IL-2_LD_. We first verified that we could indeed transplant an IL-2_LD_–tuned microbiota to naive mice (so-called IL-2_LD_–tuned fecal microbiota transplantation [FMT]). NOD mice were treated with IL-2–producing or luciferase-producing AAV vectors_,_ and their gut microbiota were collected 30 days thereafter. Collected microbiota were orally transferred in 2 groups of 6-week-old recipient NOD mice whose gut microbiota had been cleared with a cocktail of antibiotics (ATB) during the 14 days preceding FMT. 16S rRNA sequencing was performed on the recipient NOD mice after transplantation with IL-2_LD_–tuned FMT or with control FMT. Multidimensional scaling representation of gut microbiota composition showed that the microbiota from IL-2–treated NOD donors could be efficiently transplanted, as it was similar to that of recipient mice (Supplemental Figure 3). Furthermore, as shown in Figure 3A, 18 taxa were significantly different between recipients of gut microbiota from IL-2_LD_–treated or control NOD mice (i.e., after FMT). A heatmap of relative taxa abundances combined with hierarchical clustering showed a perfect separation between NOD mice transplanted with an IL-2_LD_–tuned or control gut microbiota. Six taxa were affected by both IL-2_LD_ treatment and IL-2_LD_–tuned FMT in NOD mice (Figure 3B). Of these, *Desulfovibrio,* the *Lachnospiraceae bacterium A2* species*,* an uncultured genus of the *Ruminococcaceae* family, and an uncharacterized genus of the *Clostridiales vadinBB60 group* family were upregulated or downregulated in a concordant manner in the 2 conditions.

Because an IL-2_LD_–tuned microbiota can be engrafted, we tested whether a transferred IL-2_LD_–tuned microbiota could confer some protection against autoimmunity and gut inflammation. An IL-2_LD_–tuned microbiota FMT did indeed significantly protect C57BL/6J mice from colitis induced by DSS. Weight loss (Figure 3C) and disease activity index (Figure 3D) were markedly and significantly reduced compared with untreated mice, to mice that received control FMT, and to mice treated with ATB. The mice having received ATB and no FMT behaved as the untreated control mice. This is not surprising because the colitis was induced 2 weeks after the ATB treatment, which left time for microbiota reconstitution.

Similarly, NOD mice that received IL-2_LD_–tuned microbiota had a reduction of diabetes incidence compared with mice that received control FMT in the 50 weeks after treatment (Figure 3E).

Altogether, these results showed that an IL-2_LD_–tuned microbiota could be efficiently transplanted to IL-2–naive recipient mice and protected against autoimmunity and gut inflammation in 2 murine genetic backgrounds. These results prompted us to analyze further the effects of IL-2 on gut microbiota by shotgun metagenomic sequencing.

### Identification of microbiota species affected by IL-2_LD_.

To further characterize the changes in gut microbiota composition induced by IL-2_LD_, we performed gut microbiome profiling from IL-2_LD_–treated NOD and C57BL/6J mice using shotgun metagenomics. The Simka algorithm was first used to quantify the effects of the IL-2 treatment between profiles without defining a prior metagenome reference (26).

**Figure 4:**
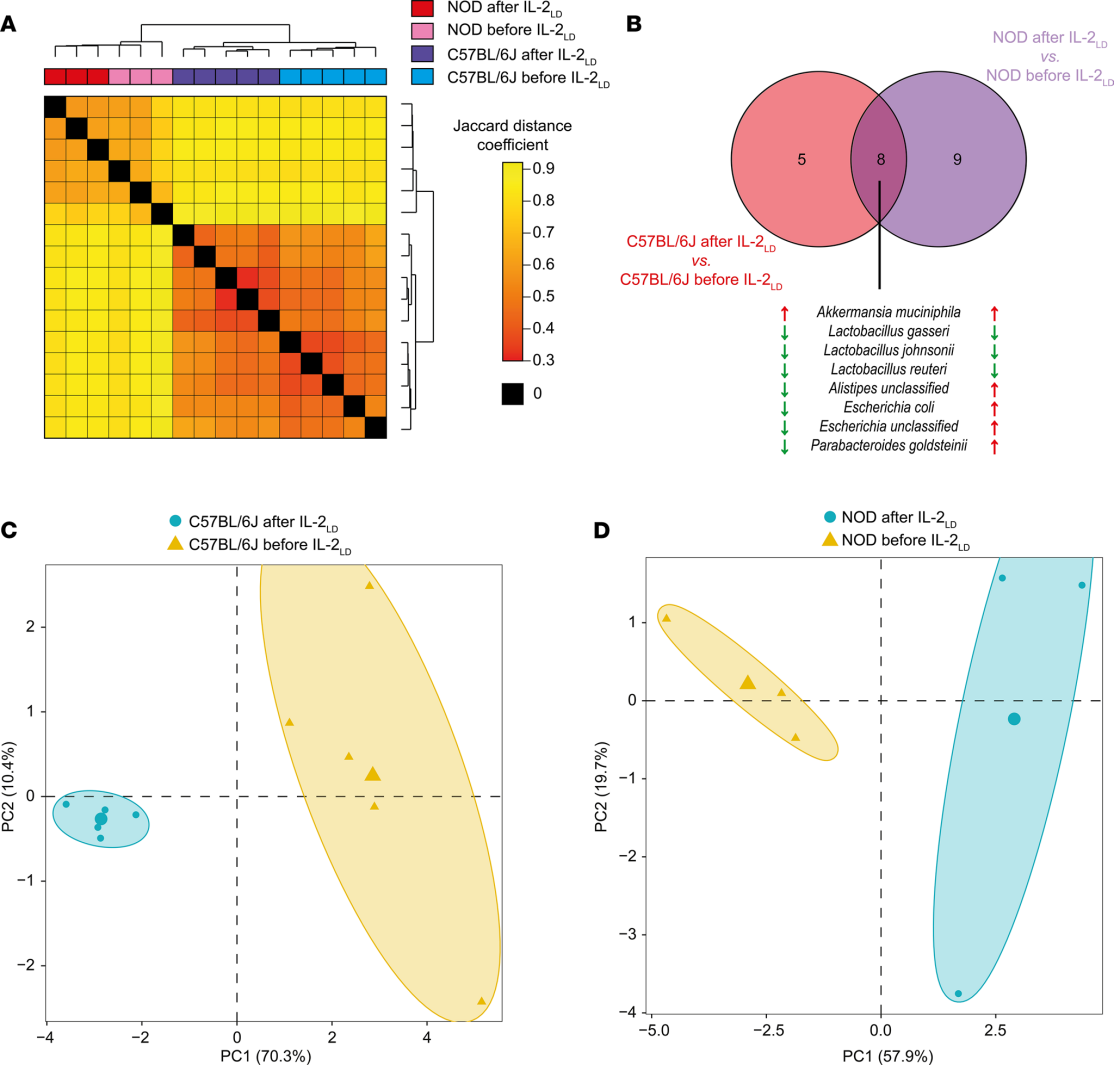
Metagenomics reveals microbial species affected by IL-2_LD_. To gain more insight into the impact of IL-2_LD_ on microbial composition, gut microbiome profiling of NOD and C57BL/6J mice before and 30 days after IL-2_LD_ treatment was performed using metagenomics. (**A**) Distogram representation showing the distance between microbiome profiles. Similarities were calculated using the Simka algorithm, which compares profiles using a k-mer approach without needing a reference metagenome, and using the Jaccard distance coefficient on abundance levels. (**B**) Venn diagram showing the overlap between the lists of taxa significantly affected by IL-2_LD_ in NOD and C57BL/6J mice. The names of the overlapping species are indicated, and their upregulation and downregulation relative to controls are respectively indicated by red or green arrows. (**C** and **D**) Principal component analysis representations of the gut microbiome profiles of NOD and C57BL/6J mice before and after treatment with IL-2_LD_ generated using the abundance levels of taxa significantly affected by IL-2_LD_. IL-2_LD_ was administered to mice by means of an IL-2–producing AAV vector.

Clustering based on the Jaccard distance perfectly separated NOD and C57BL/6J mice, and within these clusters, separated the IL-2_LD_–treated mice from the untreated mice (Figure 4A), indicating that the NOD microbiota still differed from that of C57BL/6J mice after IL-2_LD_ treatment. We found that 13 taxa were dysregulated by IL-2_LD_ in C57BL/6J mice and 17 were dysregulated in NOD mice, with 8 species simultaneously affected in the 2 mouse backgrounds (Figure 4B). *Akkermansia muciniphila* was upregulated by IL-2_LD_ in both mouse backgrounds. *Lactobacillus gasseri*, *Lactobacillus johnsonii*, and *Lactobacillus reuteri* were downregulated by IL-2_LD_ in both mouse backgrounds. Additionally, *Alistipes unclassified*, *E. coli*, *Escherichia unclassified*, and *Parabacteroides goldsteinii* were dysregulated by IL-2_LD_ but with different directionalities.

Principal component analysis based on abundances clearly showed a strong effect of IL-2_LD_ in both genetic backgrounds (Figure 4, C and D). It is noteworthy that these results were reproduced using an independent computational analysis (Supplemental Figure 4, A and B).

### Identification of microbial functional pathways affected by IL-2_LD_.

**Figure 5:**
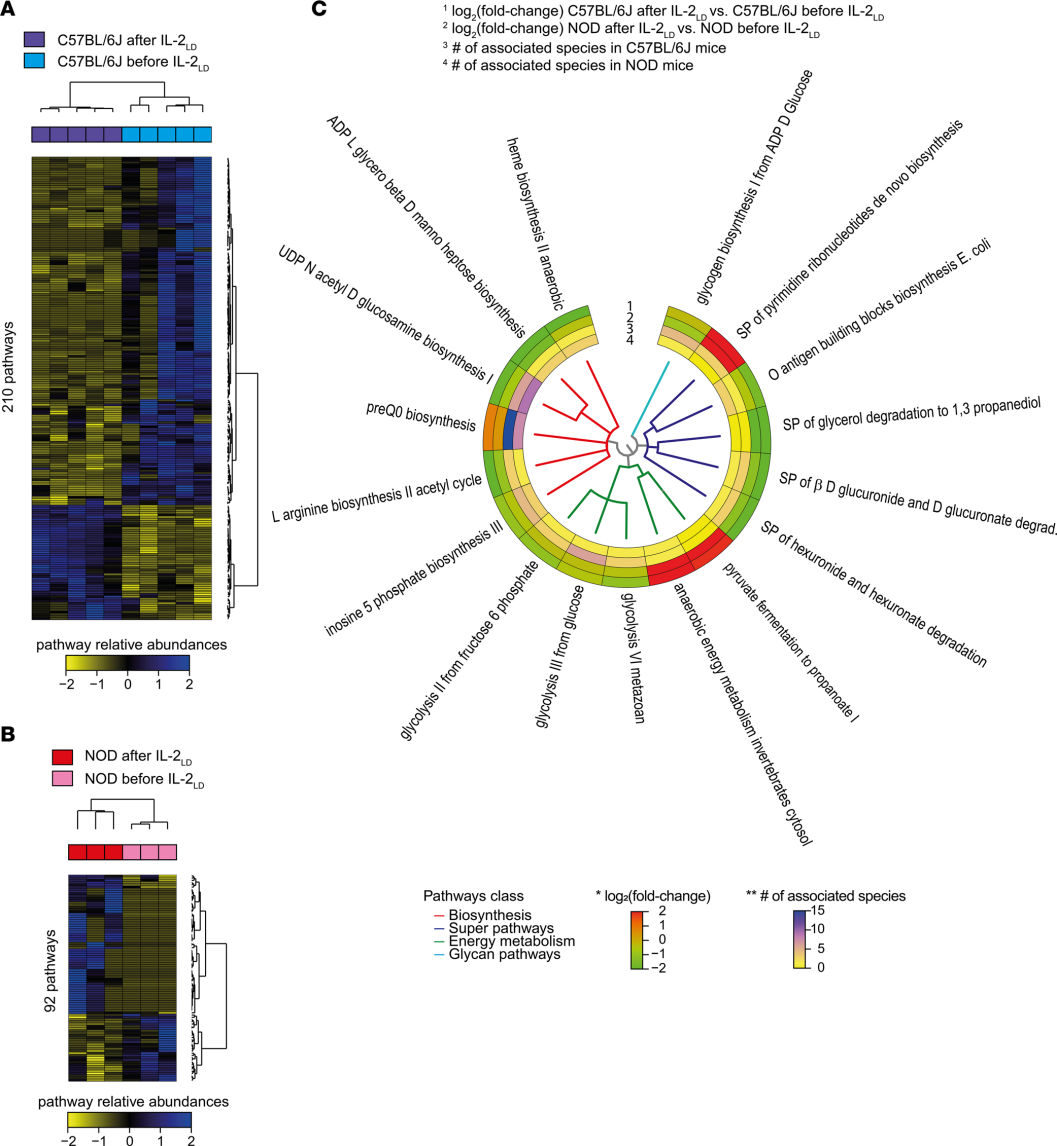
Metagenomics reveals microbial pathways affected by IL-2_LD_. (**A** and **B**) Heatmap representations combined with unsupervised hierarchical clustering of relative abundance levels for microbial pathways affected by the IL-2_LD_ treatment in C57BL/6J and NOD mice relative to their baseline conditions. (**C**) Circular tree representation using color-gradient scales showing the fold-change of microbial pathway abundances and the number of species associated for the 17 pathways affected by IL-2_LD_ in C57BL/6J and NOD mice and with the same directionality. The classes of each pathway are also indicated in different colors. The * symbol indicates the log_2_ of the fold changes of microbial pathway abundances using a color-gradient scale ranging from green to red. The ** symbol indicates the number of associated species to each pathway using a color-gradient scale ranging from yellow to blue. IL-2_LD_ was administered to mice by means of an IL-2–producing AAV vector.

We next performed a functional analysis to gain more insight into microbial changes affected by the IL-2_LD_ treatment. We found that IL-2_LD_ affected 210 pathways in C57BL/6J mice and 92 pathways in NOD mice. Heatmaps of relative pathway abundances combined with hierarchical clustering showed a perfect separation between treated and untreated mice in both genetic backgrounds (Figure 5, A and B).

A total of 17 pathways were concordantly upregulated or downregulated in both C57BL/6J and NOD mice relative to their respective untreated controls (Figure 5C). These pathways were mainly associated with biosynthesis, energy metabolism, and glycan mechanisms — including L-arginine biosynthesis and inosine 5 phosphate biosynthesis — with many species contributing to these changes.

Overall, these results indicated that IL-2_LD_–induced modifications in microbial populations profoundly affected pathways that could affect interaction with immune cells.

### IL-2_LD_ treatment affects the microbiome of patients with autoimmune diseases.

We next assessed whether IL-2_LD_ also influences gut microbiota in humans. We performed metagenomics profiling of feces samples from 6 patients with autoimmune diseases treated by IL-2_LD_ (TRANSREG trial, ClinicalTrials.gov NCT01988506) (27). Feces were collected at baseline and between 3 and 6 months after treatment initiation.

**Figure 6:**
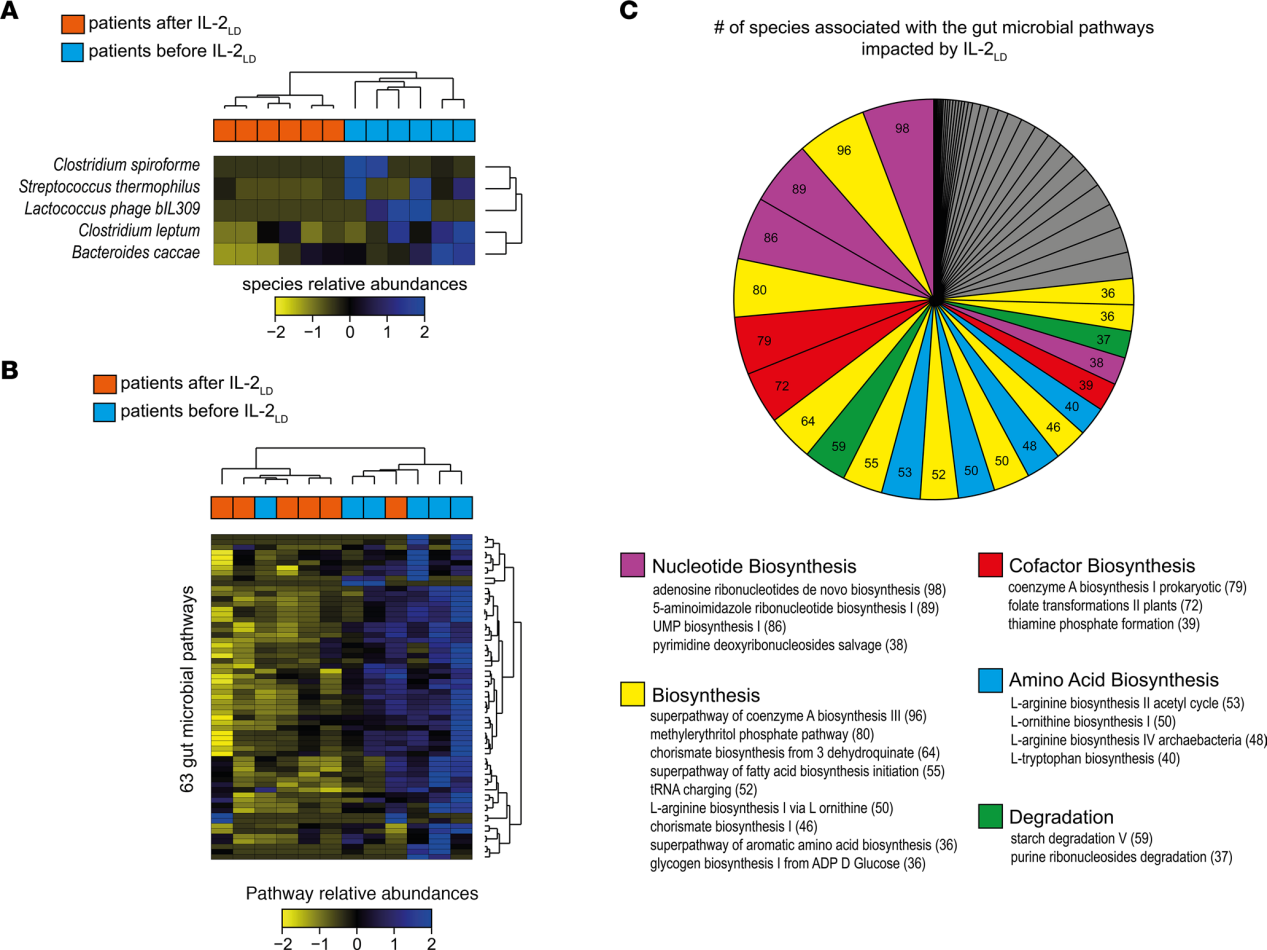
IL-2_LD_ affects the gut microbiome in patients with autoimmune disorders. To evaluate the potential of IL-2_LD_ to modify the gut microbiome in humans, 6 patients with various autoimmune diseases were treated with IL-2_LD_. Samples were collected at baseline and between 3 and 9 months after the first injection. Microbiome profiling was performed using metagenomics. (**A**) Heatmap representation of the relative abundances for the 5 bacterial species found to be differentially abundant in the gut microbiome of patients treated with IL-2_LD_ relative to the baseline condition. (**B**) Heatmap representation of relative abundances for the 63 microbial pathways found to be differentially abundant in the gut microbiome of patients. (**C**) Pie chart showing the number of taxa associated with the microbial pathways differentially abundant in patients relative to baseline. Gut microbial pathways are colored according to their classes. The number of contributing species is indicated in parentheses for each pathway. Pathways with fewer than 36 associated taxa are colored in gray.

We found 5 bacterial species affected by IL-2_LD_ in patients (Figure 6A). At the functional level, we found 63 microbial pathways affected by IL-2_LD_ in patients, all of them being downregulated in samples obtained after IL-2_LD_ treatment relative to the respective baseline samples (Figure 6B). These pathways were mainly associated with nucleotide biosynthesis, biosynthesis, cofactor biosynthesis, amino acid biosynthesis, and degradation (Figure 6C). For each of these pathways, we quantified the number of associated bacterial species (i.e., the number of species expressing each given pathway). We found that the adenosine ribonucleotide de novo biosynthesis, super pathway of coenzyme A biosynthesis III, 5-aminoimidazole ribonucleotide biosynthesis I, UMP biosynthesis I, and methylerythritol phosphate pathways had the highest numbers of associated species (>80). L-arginine biosynthesis was also found to be affected by IL-2_LD_ with multiple pathways having high numbers of associated species.

The 6 patients’ pre– and post–IL-2LD gut microbiota samples clustered well together, as shown by the heatmaps of relative taxa abundances (Figure 6A) or pathways (Figure 6B) combined with unsupervised hierarchical clustering.

## Discussion

We demonstrated here that IL-2 shaped gut microbiota, at both the taxonomic and functional levels, in 3 murine genetic backgrounds and in patients with autoimmune diseases. Moreover, we showed that IL-2–tuned microbiota could be transplanted and protected IL-2–naive recipient mice against experimental inflammatory bowel disease and diabetes.

### IL-2_LD_ shapes fecal microbiota in different species.

Gut microbiota has the potential to shape the immune system (19, 28). In return, the immune system can have an impact on gut microbiota. Recently, modulation of expression of IL-17, a proinflammatory cytokine that contributes to both autoimmunity and host immune defense, has been reported to induce modifications of gut microbiota (29) that led to protection against central nervous system autoimmunity. Mirroring these observations, we showed that a cytokine that expands and activates Tregs also shaped gut microbiota composition in 3 mouse genetic backgrounds and in humans. In mice, principal component analysis and hierarchical clustering perfectly separated the microbiota of IL-2–treated versus control mice in the 3 genetic backgrounds, using 2 independent taxonomic profiling methods. Interestingly, the number of microbial taxa that changed after IL-2_LD_ was always higher in NOD mice than in BALB/c or in C57BL/6J mice. Because NOD mice have genetically controlled low IL-2 production that leads to Treg deficiency (25), this emphasizes the importance of the gut microbiota/IL-2/Treg axis in health and disease. Our results extend the recent observation that IL-2 leads to modification of gut microbiota in NOD mice using 16S rRNA analysis (23). Here, we extend this work by showing that IL-2–tuned microbiota had an immunoregulatory effect and by analyzing IL-2–treated patients. We observed a clear impact of IL-2_LD_ on the microbiome in humans, although this impact was less pronounced than that observed in mice, likely because of higher interindividual variability. Nevertheless, as for mice, we could accurately cluster together the pre–IL-2_LD_ gut microbiota and post–IL-2_LD_ gut microbiota from these patients. This is remarkable. Indeed, given that diet as well as disease conditions profoundly affect gut microbiota composition, it could have been expected that pre– and post–IL-2_LD_ treatment samples from a given patient would have clustered together. Thus, based on the differentially abundant taxa and pathways, the robustness of the gut microbiota changes induced by IL-2_LD_ in humans was more discriminating than the individual’s gut microbiota composition.

### IL-2 licenses the gut microbiota to act as an immunomodulatory drug.

The bidirectional interdependency of the gut microbiota and the immune system has raised the possibility that gut microbiota could modulate/mediate the efficacy of immunotherapies, which has indeed been observed for cancer immunotherapies (30–32). Here, we report the ability of an immunomodulatory treatment targeting Tregs to confer its immunoregulatory potential on gut microbiota.

This is important because alternatives to IL-2–producing AAV or IL-2 intraperitoneal injections have now been developed to stimulate Tregs. Among them is the use of IL-2 complexes with enhanced specificity for Tregs (33) or the use of the superagonistic anti-CD28 antibody (34). The impact of these Treg stimulation methods on the gut microbiome has yet to be studied.

Note that IL-2–tuned FMT resulted in control of 2 independent disease conditions, like the IL-2_LD_ treatment itself. Thus, IL-2_LD_ not only changed the composition of intestinal microbial taxa, but also licensed microbiota to act as an immunomodulatory treatment. This opens the interesting possibility of manipulating the microbiota before its transplantation, so as to improve its efficacy.

### Gut microbiota modifications induced by IL-2 are relevant for the immunomodulatory effect of IL-2_LD_–tuned microbiota.

Our gut microbiota profiling provides some insights into the potential mechanisms by which IL-2_LD_ may affect the host. First, the majority of taxa differentially expressed in IL-2_LD_–treated NOD mice belong to the *Ruminococcaceae* family. Members of this family have been reported to be decreased in many autoimmune or inflammatory diseases, notably in patients with type 1 diabetes or Crohn’s disease (35, 36). Second, taxa of *Intestinimonas*, the most differentially abundant genus in IL-2_LD_–treated-NOD mice compared with controls, have been reported to be decreased in patients with type 1 diabetes (35). *Intestinimonas* are also known to be producers of short-chain fatty acids, which are known to promote immunomodulation by favoring Tregs and limiting effector T cells (37–39). Third, *Akkermansia muciniphila* is increased by IL-2_LD_ treatment in NOD and C57BL/6J mice and is more differentially abundant in IL-2–treated NOD mice than in IL-2–treated C57BL/6J mice. This taxon a) has been described as a protector against autoimmunity in NOD mice and against DSS-induced colitis, and b) can also promote Tregs in inflammatory bowel diseases in mice (40, 41). Furthermore, the abundance of *Akkermansia muciniphila* has been shown to be associated with Treg proliferation (42). Fourth, Rowan et al. found that *Desulfovibrio* was significantly increased in acute and chronic human ulcerative colitis (43). Here, we found that this species was increased in IL-2_LD_–treated NOD mice compared with untreated NOD mice, as well as in NOD mice with IL-2_LD_–tuned FMT compared with NOD mice with control FMT. Additionally, the *Desulfovibrio species* was found to be upregulated in untreated NOD mice relative to untreated BALB/c mice.

Together, these observations point to a crosstalk between Tregs and these taxa that could provide at least part of the mechanism driving the immunoregulatory efficacy of FMT of IL-2–tuned microbiota.

Downstream of the modifications of taxa composition by IL-2_LD_, we also identified functional pathways that could participate in the immunoregulatory efficacy of IL-2–tuned microbiota. We identified a core of 17 microbial metabolic pathways that changed in both NOD and C57BL/6J mice after this IL-2_LD_ treatment. The biosynthesis of L-arginine, an amino acid described as a fuel for the generation of citrullinated peptides that could trigger autoimmune responses, notably in type 1 diabetes (44–46), is decreased in intestinal microbes of IL-2_LD_–treated mice and humans. Conversely, metabolic pathways leading to the increase of short-chain fatty acids (e.g., pyruvate fermentation to propanoate I) were increased by IL-2_LD_ treatment in both murine genetic backgrounds. This observation is in line with our previous comment related to the major increase in *Intestinimonas,* known as short-chain fatty acid producers, in IL-2_LD_–treated NOD mice. With the recent report that FMT from healthy donors halts the progression of new-onset type 1 diabetes in humans (47), our results suggest that the use of IL-2–tuned FMT or of FMT enriched in specific taxa could represent an improved treatment modality.

In summary, gut microbiota modifications induced by IL-2_LD_ appear to favor taxa and microbe metabolic pathways that influence the Treg/effector T cell balance. Our results call for extended studies in human cohorts to confirm our observations. Our work suggests a virtuous circle in which IL-2_LD_ affects gut microbiota, and in return gut microbiota reinforces the primary effect of IL-2_LD_ on Treg/effector T cell balance. It should be noted that Tregs can shape gut microbiota (48), and it is also known that IL-2_LD_ directly affects Tregs. Thus, we can postulate that IL-2 may affect gut microbiota through the modulation of Tregs. This has implications for a better understanding of how gut microbiota participates in and maintains disease conditions and for the better design of immunoregulatory therapies, including improved FMT.

The fact that IL-2–tuned microbiota act as an immunomodulatory treatment opens the possibility to manipulate gut microbiota for improved FMT and to discover microbial-derived molecules to treat autoimmune and inflammatory diseases. The effects of IL-2_LD_–tuned FMT on the recipient immune system, notably at the level of the T cell receptor (TCR) repertoire of Tregs, have yet to be evaluated.

## Methods

### Housing of mice and ethical statements.

Female BALB/c mice, C57BL/6J mice, NOD mice, and NOD mice expressing GFP under the control Foxp3 gene promoter were maintained under pathogen-free conditions according to European legislation. All mice received the same food, which was normal-protein chow.

### Administration of IL-2_LD_.

IL-2_LD_ was administered either a) by a single injection of an IL-2–producing recombinant AAV vector serotype 8 (AAV8) (rAAV) vector and compared with the injection of a luciferase-producing control rAAV, both at a concentration of 10^10^ rAAV viral genomes as previously described (24); or b) by intraperitoneal injections with 50,000 units of ILT-101 (human recombinant IL-2; ILTOO Pharma) during 5 consecutive days and compared with an injection of PBS.

### Cytometry profiling.

Cells were collected from NOD Foxp3 GFP mice. Blood was submitted to RBC lysis. Pancreas was digested with collagenase/DNase solution and filtered as previously described (49). Lamina propria cells were obtained with the Lamina Propria Dissociation kit and the GentleMACS Octo Dissociator with heaters (Miltenyi Biotec). For lamina propria, a Percoll density gradient step was performed as previously described (49). We used the following monoclonal antibodies at predetermined optimal dilutions for 20 minutes at 4°C: Live Dead eFluor 780 (Thermo Fisher Scientific; catalog 65-0865-18), CD3 eFluor 450 (Thermo Fisher Scientific; clone 17A2; catalog 48-0032-82), CD8 Super Bright 600 (Thermo Fisher Scientific; clone RPA-T8; catalog 63-0088-42), CD4 Horizon v500 (BD Biosciences; clone RM4-5; catalog 560782), CD45 PE-CF594 (BD Biosciences; clone 30-F11; catalog 562420), and NKp46 Alexa Fluor 700 (BD Biosciences; clone 29A1.4; catalog 561169). Cells were acquired on a Cytoflex LX flow cytometer (Beckman Coulter) and analyzed using FlowJo software. Tregs were defined as GFP^+^ cells among CD4^+^ cells.

### Monitoring of diabetes in NOD mice.

Urinary glucose was measured every 2 days using colorimetric strips (Multistix, Bayer), and blood glucose was quantified by a glucometer (Optium Xceed, Abbott Diabetes Care). NOD mice were considered diabetic after 2 consecutive blood glucose readings above 250 mg/dL. In our laboratory conditions, there is no reversal of diabetes after 2 such consecutive hyperglycemic readings (49), and mice have the classic accompanying histology of islet destruction. Note that the incidence of diabetes in NOD mice is known to vary depending on the cleanliness of the animal facilities.

### Induction and monitoring of DSS-induced colitis in C57BL/6J mice.

DSS was added to drinking water on day 0 (50). We used a concentration of 2% DSS in water. Mice were monitored 3 times a week for body weight, stool consistency, and the presence of blood in the stool (disease activity index assessment).

### FMT.

Before FMT, female mice were orally gavage-fed with 200 μL of a combination of metronidazole (1 g/L), vancomycin (500 mg/L), ampicillin (1 g/L), and neomycin sulfate (1 g/L) in sterile water for 14 days as previously described (51, 52). Then, 200 mg of fresh stool (10 pellets) was suspended in 50 volumes of sterile water (1 mL) and 400 μL of this suspension was given to each recipient mouse by oral gavage using a 24G round tip gavage needle, for 2 consecutive days (53).

### Mouse fecal DNA extraction and 16S rRNA gene sequencing.

Mouse fecal genomic DNA was extracted and 16S rRNA sequenced as previously described (54).

### Processing of 16S rRNA gene sequencing data.

Raw-read quality filtering was performed with the PRINSEQ-lite (55) using a Phred quality threshold of 30. Paired-end reads were assembled using FLASH with a minimum overlap of 30 bases and a 97% overlap identity (56). Finally, CutAdapt was used to remove both forward and reverse primer sequences, with no mismatches allowed in those sequences (57).

### Analysis of 16S rRNA sequencing data.

Microbiota profiles were analyzed using QIIME2 based on the SILVA (release 132) database (58). Taxa with a cumulated abundance lower than 0.01% in the whole data set were filtered out. Differentially abundant taxa were identified using a sparse partial least-squares discriminant analysis (sPLS-DA) approach with a feature selection based on an effect size for the Wilcoxon signed-rank test higher than 0.5.

### Collection of human samples.

Six patients from the TRANSREG trial (ClinicalTrials.gov NCT01988506) (27) (2 with Sjögren’s disease, 1 with Crohn’s disease, 1 with Behçet’s disease, 1 with psoriasis, and 1 with systemic sclerosis) had available feces samples collected at baseline and between 3 and 6 months after the first IL-2_LD_ injection.

### Human and mouse fecal metagenomic profiling.

Total fecal DNA was shotgun-sequenced using ion-proton technology (Thermo Fisher Scientific), resulting in 23.7 ± 0.8 million (mean ± SD) single-end short reads of 150 bases as a mean. Each sample produced at least 20 million single-end DNA reads from 45 to 370 bp.

### Processing of metagenomic sequencing data.

Raw reads were cleaned using AlienTrimmer to remove resilient sequencing adapters and low-quality bases (59). Reads were filtered from human DNA and other possible food contaminant DNA (using *Homo sapiens*, *Bos taurus*, and *Arabidopsis thaliana* assemblies and an identity score threshold of 97%). Reads with a length less than 100 bp were removed.

### Analysis of metagenomic sequencing data.

MetaPhlAn2 and HUMAnN2 were used to assess the relative abundance of microbial communities and microbial pathways (60, 61). Taxa with an accumulated abundance lower than 0.01% in the whole data set were filtered out. Species and pathways differentially abundant between conditions were identified using an sPLS-DA approach (62). Prior to the discriminant analysis, a feature selection was performed to select species and pathways with an effect size for a Wilcoxon signed-rank test higher than 0.5 for patients.

### Confirmatory principal component analysis of mouse metagenomic profiles.

The confirmatory principal component analysis was generated as described below. A gut microbiome gene catalog was built using bacterial genes extracted from proprietary Illumina sequencing data of the gut microbiome from selected mouse strains (HDDR1, Taconix, BALB/c, and C57BL/6J) and the Illumina sequencing data from the public mouse microbiome gene catalog (63). This gut microbiome gene catalog was built by performing metagenomic assembly using MegaHit (v1.1.2) followed by gene prediction using GeneMark-HM (64) and by clustering all the gene sequences at 95% identity over 90% length coverage using CD-HIT (v4.7). All the sequencing data used for these steps were mapped back on the microbiome gene catalog using BBmap, retaining only reads aligned with at least 95% identity and with a minimum length of 45 nucleotides. An abundance table was derived using the raw-read counts of each gene. This abundance information was used by MSPminer to generate 761 metagenomic species pangenomes (MSPs) (65).

High-quality sequencing data from the mouse strains analyzed in the present work were aligned against this newly constructed mouse gut microbiome gene catalog using BBmap with the same parameters as described above. Raw-read gene counts were normalized by the gene length and scaled by the total number of reads per sample to generate relative abundance values for each gene. MSP abundances were calculated by summing all the relative abundances of each gene belonging to a particular MSP and analyzed using R (v3.5) to perform the different statistical analyses. Principal component analysis was performed using the centered and scaled abundance values of the MSPs that were present in at least 70% of the samples (674 MSPs for NOD mice and 643 MSPs for C57BL/6J mice), using the prcomp function.

### Data and materials availability.

16S rRNA gene sequencing and shotgun metagenomics sequencing data have been deposited in NCBI’s Sequence Read Archive (SRA) database and are available through the accession number PRJNA831236.

### Statistics.

The 2-tailed Student’s *t* test was used to compare body weight variation, disease activity, and Treg abundance in treated and control mice at each time point. The χ^2^ test was used to compare the Kaplan-Meier estimators. The Holm-Bonferroni adjustment was used for multiple-comparison correction. A *P* value less than 0.05 was considered significant.

### Study approval.

All animal procedures were approved by the Regional Ethics Committee on Animal Experimentation No. 5 of the Ile-de-France region (Ce5/2012/031). All participants provided written informed consent to participate in the study. The human study was approved by the IRB of the Pitié-Salpêtrière Hospital (CPP-IdFVI) and performed in accordance with the Declaration of Helsinki and good clinical practice.

## Author contributions

DK contributed to the study concept, supervision, and funding. JC, GC, and GF conducted mouse experiments. TWH and HS performed 16S rRNA sequencing. ELC and SDE conducted metagenomics shotgun sequencing. All authors contributed to data analysis. NT, ONT, GP, SD, and DK wrote the manuscript. FS, CG, and CB performed the confirmatory principal component analysis of mouse metagenomic profiles. All authors revised the manuscript and approved its final version.

## Supplementary Material

Supplemental data

## Version 1 08/02/2022

In-Press Preview

## Version 2 09/08/2022

Electronic publication
