# Supplementary material for: Low-dose IL-2 shapes a tolerogenic gut microbiota that improves autoimmunity and gut inflammation
Source: JCI Insight. 2022 Sep 8;7(17):e159406. doi: 10.1172/jci.insight.159406 (PMC9536277; doi:10.1172/jci.insight.159406)
Supplement: Supplemental data [file jciinsight-7-159406-s197.pdf]

Low-dose interleukin-2 shapes a tolerogenic gut microbiota that improves autoimmunity and gut inflammation.

Supplementary Materials

Nicolas Tchitchek[1], Otriv Nguekap Tchoumba[1,2†], Gabriel Pires[1†], Sarah Dandou[1], Julien Campagne[1], Guillaume Churlaud[1,2], Gwladys Fourcade[1], Thomas W. Hoffmann[3], Francesco Strozzi[4], Camille Gaal[4], Christophe Bonny[4], Emmanuelle Le Chatelier[5], Dusko Erlich[5], Harry Sokol[3,6,7] and David Klatzmann[1,2*]

[1]Sorbonne Université, INSERM, Immunology-Immunopathology-Immunotherapy (i3), F-75005 Paris, France.

[2]AP-HP, Hôpital Pitié-Salpêtrière, Biotherapy (CIC-BTi) and Inflammation-Immunopathology-Biotherapy Department (i2B), F-75013, Paris, France.

[3]Micalis Institute, Institut National de la Recherche Agronomique (INRA), AgroParisTech, Univ Paris-Saclay ; F-78352, Jouy-en-Josas, France.

Sorbonne University-UPMC Univ Paris 06, INSERM ERL 1157, Avenir Team Gut Microbiota and Immunity, UMR 7203 ; F-75005, Paris, France.

[4]Enterome, 94/96 Avenue Ledru-Rollin, 75011 Paris, France

[5]MetaGenoPolis, INRA, Université Paris-Saclay, 78350, Jouy-en-Josas, France.

[6]Sorbonne Université, Ecole Normale Supérieure, CNRS, INSERM, AP-HP, Laboratoires des Biomolécules (LBM) ; F-75012, Paris, France.

[7]AP-HP, Hôpital Saint Antoine, Department of Gastroenterology and Inflammation-Immunopathology-Biotherapy Department (i2B), F-75011, Paris, France.

[†] These authors contributed equally to this work

[*] Corresponding author. Email: david.klatzmann@sorbonne-universite.fr

SUPPLEMENTARY FIGURES

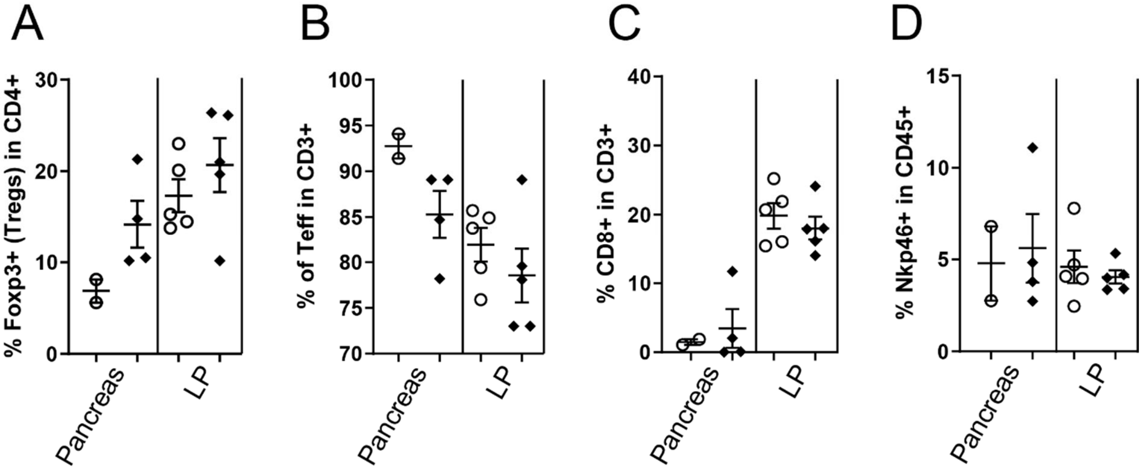

Supplementary Figure 1 – Distribution of Tregs, Teffs, CD8$^+$, and NK cells in organs of NOD mice treated with IL2LD or not. The percentages of (**A**) Foxp3+ cells among CD4$^+$ cells, (**B**) Teffs among CD3$^+$ cells, (**C**) CD8+ among CD3$^+$ cells, and (**D**) Nkp46$^+$ among CD45$^+$ cells were quantified in the pancreas and intestinal lamina propria (LP) of NOD mice injected with IL2-producing AAV (in black) or untreated (in white). Statistical significances are reported as follows: * p-value < 0.05; ** p-value < 0.01; *** p-value < 0.001.

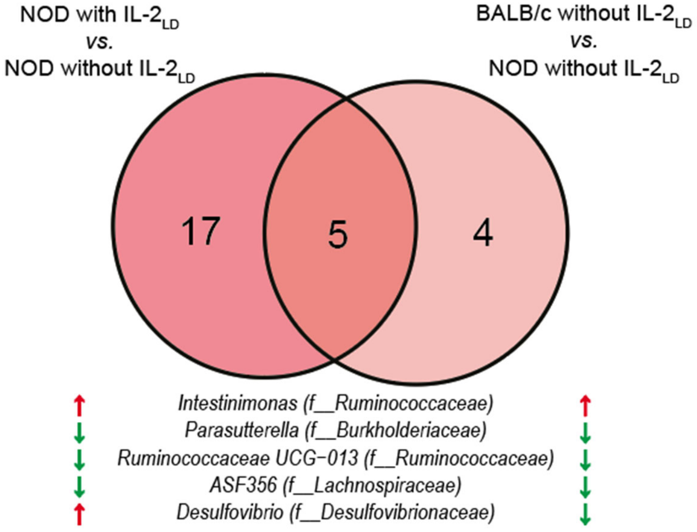

Supplementary Figure 2 – Venn diagram showing the overlap between the lists of taxa impacted by IL-2_{LD} in NOD mice and the taxa naturally differentially abundant in BALB mice relative to NOD mice. The names of the overlapping species are indicated, and their upregulation and down-regulation relative to controls are indicated by red or green arrows.

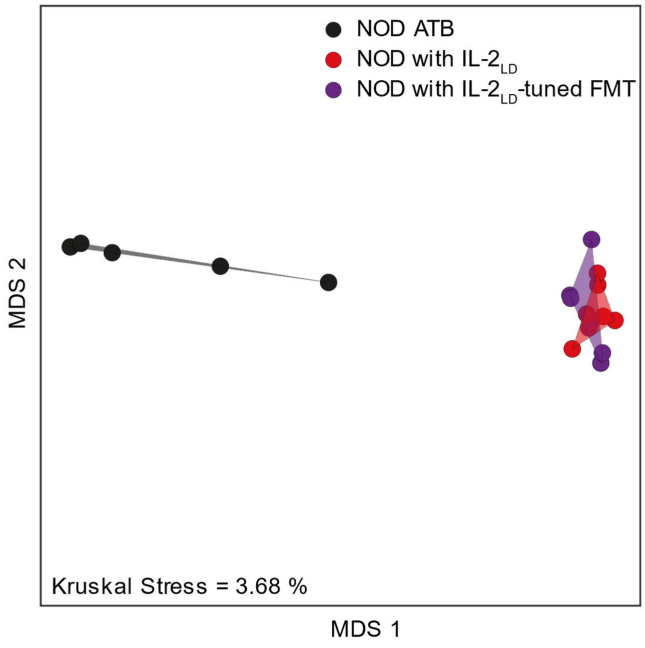

Supplementary Figure 3 – Multidimensional scaling representation of gut microbiota profiles of NOD mice treated only with antibiotics (black), treated with IL-2_{LD} (red), or receiving IL-2_{LD}-tuned FMT (purple). Each dot in the representation corresponds to a microbiota profile obtained from 16S rRNA sequencing, and the dots are positioned according to their similarities computed based on taxa abundance levels. The Kruskal Stress indicated at the bottom of the representation quantifies the quality of the representation as the fraction of information lost during the dimensionality reduction process.

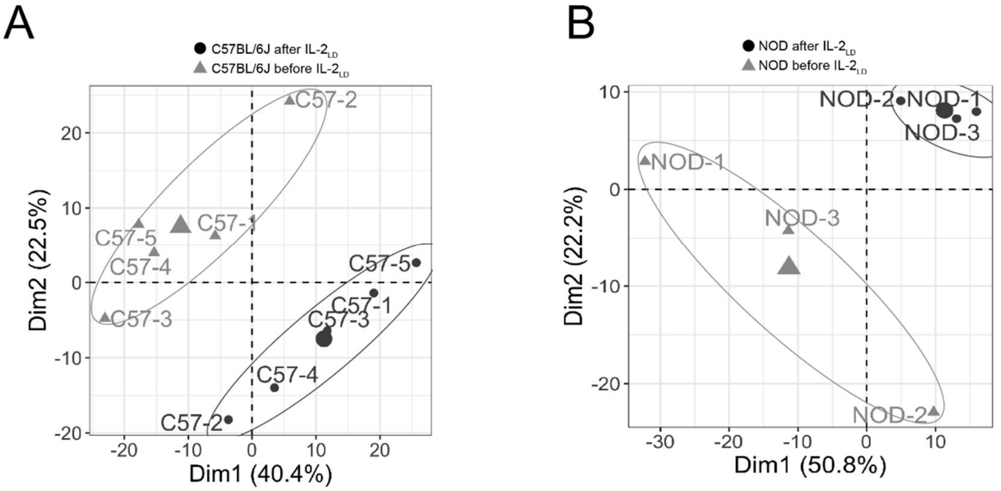

Supplementary Figure 4 – Principal component analysis representations performed using an independent analysis of the gut microbiome profiles of C57BL/6J (**A**) and NOD (**B**) mice treated or not by IL-2_{LD} generated using the abundance levels of taxa significantly impacted by IL-2_{LD}.
